# C/EBPδ protects from radiation-induced intestinal injury and sepsis by suppression of inflammatory and nitrosative stress

**DOI:** 10.1038/s41598-019-49437-x

**Published:** 2019-09-27

**Authors:** Sudip Banerjee, Qiang Fu, Sumit K. Shah, Stepan B Melnyk, Esta Sterneck, Martin Hauer-Jensen, Snehalata A. Pawar

**Affiliations:** 1 Division of Radiation Health, Department of Pharmaceutical Sciences, College of Pharmacy, University of Arkansas for Medical Sciences, Little Rock, AR 72205 USA; 2 Arkansas Children’s Research Institute, Little Rock, AR 72202 USA; 3 Laboratory of Cell and Developmental Signaling, Center for Cancer Research, National Cancer Institute, Frederick, MD 21702 USA

**Keywords:** Sepsis, Sepsis

## Abstract

Ionizing radiation (IR)-induced intestinal damage is characterized by a loss of intestinal crypt cells, intestinal barrier disruption and translocation of intestinal microflora resulting in sepsis-mediated lethality. We have shown that mice lacking C/EBPδ display IR-induced intestinal and hematopoietic injury and lethality. The purpose of this study was to investigate whether increased IR-induced inflammatory, oxidative and nitrosative stress promote intestinal injury and sepsis-mediated lethality in *Cebpd*^−/−^ mice. We found that irradiated *Cebpd*^−/−^ mice show decreased villous height, crypt depth, crypt to villi ratio and expression of the proliferation marker, proliferating cell nuclear antigen, indicative of intestinal injury. *Cebpd*^−/−^ mice show increased expression of the pro-inflammatory cytokines (*Il-6, Tnf-α*) and chemokines (*Cxcl1*, *Mcp-1*, *Mif-1α*) and *Nos2* in the intestinal tissues compared to *Cebpd*^+/+^ mice after exposure to TBI. *Cebpd*^−/−^ mice show decreased GSH/GSSG ratio, increased S-nitrosoglutathione and 3-nitrotyrosine in the intestine indicative of basal oxidative and nitrosative stress, which was exacerbated by IR. Irradiated *Cebpd*-deficient mice showed upregulation of Claudin-2 that correlated with increased intestinal permeability, presence of plasma endotoxin and bacterial translocation to the liver. Overall these results uncover a novel role for C/EBPδ in protection against IR-induced intestinal injury by suppressing inflammation and nitrosative stress and underlying sepsis-induced lethality.

## Introduction

The likelihood of civilians as well as military forces encountering radiological hazard has increased many folds with proliferation of radioactive material, nuclear weapons and nuclear power facilities^1–3^. A major side-effect of exposure to whole body radiation such as explosion of a nuclear bomb or during a nuclear accident or during abdominal radiotherapy is the acute toxicity of IR to the rapidly renewing cell systems such as the bone marrow and gastrointestinal tract mucosa^3–5^. There is a paucity of safe and effective interventions to treat or prevent IR-induced gut-associated sepsis^6–8^. In order to develop therapeutic interventions, therefore it is essential to understand the molecular underpinnings of IR-induced GI syndrome and associated lethality.

The gut mucosa is particularly radiation sensitive because of a high mucosal turnover rate^9^. The production of reactive oxygen species (ROS) and reactive nitrogen species (RNS), induced by IR promotes the induction of apoptosis and clonogenic cell death, which leads to mucosal breakdown^10–15^. It is known that exposure to IR leads to increased inflammation and that uncontrolled inflammation is known to exacerbate damage/injury to the tissues^13–15^. The process of inflammation is amplified by recruitment of neutrophils and transmigration of monocytes and activation of resident mast cells producing pro-inflammatory mediators like IL-1β, IL-6, CXCL1 and TNF-α^11–14^. This leads to the upregulation of pro-inflammatory cytokines, chemokines, and growth factors in the microvascular and mucosal compartments, presumably not only by recruited immune cells but also by enterocytes, depending on the severity of tissue trauma^14,15^.

Exposure to high doses of IR causes damage to the intestinal epithelial barrier, vascular leakage and translocation of the intestinal microflora in the blood leading to an inflammatory cascade resulting in sepsis-induced mortality and is a hallmark of the GI syndrome^16^. Although the GI syndrome and associated sepsis have been extensively characterized^17,18^, the key signaling factors that modulate IR-induced intestinal injury are not well understood.

C/EBPδ is a basic leucine zipper transcription factor that is shown to regulate target genes involved in diverse biological functions such as apoptosis, genomic instability, cell cycle, oxidative stress, stem cell self-renewal and tumor suppression^19–27^. C/EBPδ also plays an important role in regulation of the inflammatory and stress responses as well as in innate and adaptive immune response^28–31^.

We have previously shown that C/EBPδ-deficiency in mice leads to IR-induced lethality due to the underlying injury to the intestine and hematopoietic tissues^32^. Our recent studies revealed that the increased sensitivity of *Cebpd*^−/−^ mouse embryonic fibroblasts (MEFs) to IR occurs due to an impaired ability to modulate IR-induced oxidative stress and mitochondrial dysfunction^33^. Interestingly, very little is known about the exact mechanism via which C/EBPδ protects from IR-induced intestinal injury and underlying sepsis. In this study, we further investigated whether the increased IR-induced intestinal injury in *Cebpd*^−/−^ mice occurs due to an impaired ability to regulate inflammation and oxidative as well as nitrosative stress responses.

## Results

### *Cebpd*^−/−^ mice show increased intestinal injury in response to increasing doses of IR

**Figure 1:**
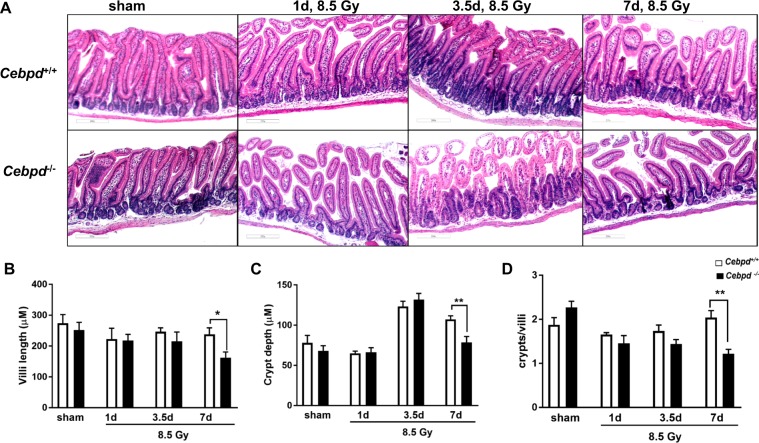
*Cebpd*−/− mice show increased damage to intestinal crypts and villi after exposure to IR. (**A**) Representative images showing the cross sections of the small intestine from *Cebpd*^+/+^ and *Cebpd*^−/−^ mice exposed to 0 and 8.5 Gy and harvested at indicated timepoints. Histograms showing the measurements of intestinal injury parameters such as (**B**) villous height, (**C**) crypt depth and (**D**) crypt to villous ratio. The data are presented as average + standard error mean (S.E.M.) of n = 4–5 mice per genotype per group.

**Figure 2:**
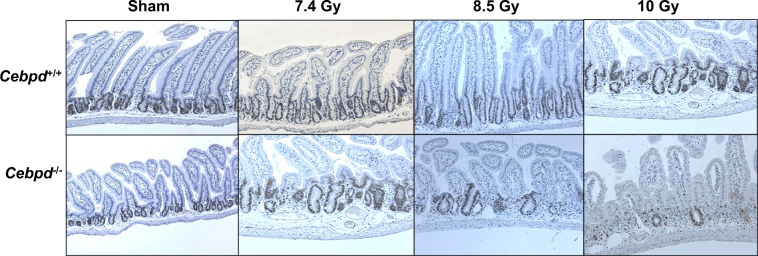
*Cebpd*^−/−^ crypts show decreased proliferation of intestinal crypts with increasing doses of radiation. Representative images showing the expression of PCNA in cross sections of the small intestine from *Cebpd*^+/+^ and *Cebpd*−/− mice exposed to 0, 7.4, 8.5 and 10 Gy and harvested at day 3.5 post-irradiation.

We have previously shown that compared to 40% mortality in *Cebpd*^+*/+*^ mice, 100% of *Cebpd*^−/−^ mice die by day 9–13 after exposure to a TBI dose of 8.5 Gy, which occurs due to the increased cell death of the intestinal stem cells of the crypts as well as injury to the bone marrow^32^. We further characterized the effects of IR exposure on the intestinal injury parameters such as villous height, crypt depth and crypt to villous ratio in the sham and irradiated *Cebpd*^+/+^ and *Cebpd*^−/−^ intestines at 1, 3.5 and 7 days post exposure to a dose of 8.5 Gy. There were no significant differences between *Cebpd*^+/+^ and *Cebpd*^−/−^ mice with respect to villous height, crypt depth and or crypts to villi ratio in the sham group and in the irradiated groups at days 1 and 3.5 post-8.5 Gy exposure. However, at day 7 post-irradiation, *Cebpd*^−/−^ mice showed significant decreases in villous height, crypt depth as well as crypt to villi ratio compared to respective *Cebpd*^+/+^ mice (Fig. 1A–C). Further, analysis of the proliferation marker -proliferating cell nuclear antigen (PCNA) revealed no significant difference between *Cebpd*^+/+^ and *Cebpd*^−/−^ intestines in the sham group. However, *Cebpd*^−/−^ mice showed fewer PCNA-positive proliferating crypts compared to *Cebpd*^+/+^ *mice* at day 3.5 post-irradiation doses of 7.4 (sublethal dose), 8.5 (LD_50/30_ dose) and 10 Gy (Fig. 2). These results suggest C/ΕΒPδ may have a protective function in the intestinal epithelial and crypt cells in the context of radiation-induced damage.

### *Cebpd*^−/−^ mice display upregulation of pro-inflammatory cytokines in intestine tissues at early timepoints after exposure to IR

It is known that exposure to IR leads to increased inflammation and that uncontrolled inflammation exacerbates damage/injury to the tissues^11,12^. We therefore compared the changes in expression of pro-inflammatory cytokines in the intestine tissues of *Cebpd*^−/−^ and *Cebpd*^+/+^ mice at various timepoints after exposure to IR.

**Figure 3:**
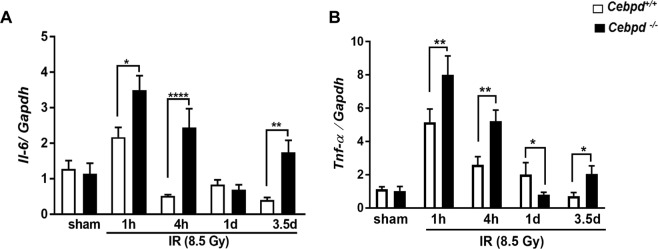
*Cebpd*^−/−^ mice display increased pro-inflammatory cytokines and chemokine expression in intestine tissues after exposure to IR. The mRNA expression of (**A**) *Il-6* and (**B**) *Tnf-α*, were analyzed in the intestines of *Cebpd*^+/+^ and *Cebpd*^−/−^ mice and expressed as fold change relative to unirradiated *Cebpd*^+/+^ mice at the indicated timepoints post-irradiation. The data are presented as average + standard error mean (S.E.M.) of n = 3–8 mice per treatment per genotype.

We found that both *Cebpd*^+/+^ mice and *Cebpd*^−/−^ mice showed IR-induced upregulation of *Il-6*. However, *Cebpd*^−/−^ mice showed significantly elevated expression of *Il-6* at 1 h (3.5-fold) and 4 h (2.4-fold) post-irradiation compared to *Cebpd*^+/+^ mice (Fig. 3A). The expression of *Il-6* was downregulated in both genotypes by day 1 post-irradiation. By day 3.5 post-irradiation, the expression of *Il-6* was 1.5-fold in *Cebpd*^−/−^ mice, while it was further downregulated in *Cebpd*^+/+^ mice, compared to respective sham controls (Fig. 3A).

Similarly, we did not find any changes in the expression of *Tnf-α* in sham mice of both genotypes, however post-irradiation there was a robust induction of *Tnf-α* in both *Cebpd*^+/+^ and *Cebpd*^−/−^ mice at 1 h and 4 h post-irradiation. *Cebpd*^+/+^ mice showed a 5-fold induction, while *Cebpd*^−/−^ mice showed 8-fold induction of *Tnf-α* at 1 h post-irradiation compared to respective sham groups. *Cebpd*^+/+^ mice showed 2.5-fold induction at 4 h post-irradiation and about 2-fold at day 1 post-irradiation. In contrast, while *Cebpd*^−/−^ mice showed 5-fold induction of *Tnf-α* at 4 h post-irradiation, but was rapidly downregulated to about 0.5-fold compared to the respective sham group at day 1 post-irradiation (Fig. 3B). Overall, these results indicate elevated expression of the inflammatory cytokines *Il-6* and *Tnf-α* in *Cebpd*^−/−^ mice compared to *Cebpd*^+/+^ mice.

### *Cebpd*^−/−^ showed elevated expression of chemokines post-irradiation compared to *Cebpd*^+/+^ mice

**Figure 4:**
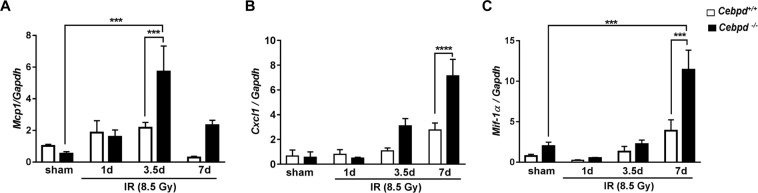
*Cebpd*^−/−^ mice increased intestinal expression of chemokines after exposure to IR. The mRNA expression of (**A**) *Mcp-1*, (**B**) *Cxcl1* and (**C**) *Mif-1-α*, were analyzed in the intestines of Cebpd^+/+^ and *Cebpd*^−/−^ mice and expressed as fold change relative to unirradiated *Cebpd*^+/+^ mice at the indicated timepoints post-irradiation. The data are presented as average + S.E.M. of n = 3–8 mice per treatment per genotype.

Next, we examined the expression levels of chemokines, which play a prominent role in the recruitment of inflammatory cells to damaged tissues. Monocyte chemoattractant protein-1 (Mcp-1) is a chemokine that recruits monocytes and macrophages to the sites of inflammation^34^. The expression of *Mcp-1* was upregulated in both *Cebpd*^+/+^ and *Cebpd*^−/−^ mice at day 1 post-irradiation by about 2-fold compared to respective shams. However, by day 3.5 post-irradiation, *Cebpd*^−/−^ mice showed a 6-fold increase in the expression of *Mcp-1* compared to *Cepbd*^+/+^ mice (Fig. 4A). In contrast, at day 7 post-irradiation *Cebpd*^−/−^ mice showed a 1.8-fold increase, while *Cebpd*^+/+^ mice showed a dramatic decrease to 0.24-fold when compared with the respective sham groups (Fig. 4A).

*Cxcl1* {Chemokine (C-X-C motif) ligand 1} also known as KC is another chemokine expressed by macrophages, neutrophils and epithelial cells and has neutrophil chemoattractant activity^35^. There were no significant differences in the intestinal expression of *Cxcl1* between both the genotypes in unirradiated mice. At day 7 post-irradiation. *Cebpd*^−/−^ mice showed a 7-fold increase, while *Cebpd*^+/+^ mice showed a 2-fold increase in the expression of *Cxcl1* in the intestine tissues (Fig. 4B).

Macrophage migration inhibitory factor (*Mif1-α*), a pro-inflammatory cytokine which is upregulated by IR and oxidative stress^36^. MIF also has a chemokine-like function and promotes the directed migration and recruitment of leukocytes into infectious and inflammatory sites^37,38^. Unirradiated *Cebpd*^−/−^ mice showed slightly higher expression of *Mif-1α*, however this difference was not significant when compared with unirradiated *Cebpd*^+/+^ mice. Interestingly at day 7 post-irradiation, *Cebpd*^+/+^ mice showed a 2-fold induction, while *Cebpd*^−/−^ mice showed a robust upregulation of *Mif-1α* by almost 10-fold (Fig. 4C).

These results indicate that the elevated expression of chemokines may promote the increased recruitment of neutrophils and macrophages, in the intestine tissues of *Cebpd*^−/−^ mice compared to that of *Cebpd*^+/+^ mice after exposure to IR.

### *Cebpd*-deficiency promotes increased nitrosative stress in the intestine prior to and post-irradiation

**Figure 5:**
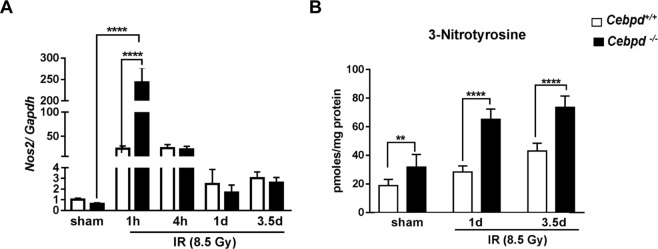
*Cebpd*^−/−^ mice show increased nitrosative stress prior to and after exposure to IR. (**A**) The mRNA expression of *Nos2* was analyzed and expressed as fold changes relative to that of unirradiated *Cebpd*^+/+^ mice and presented as an average + S.E.M of n = 4–9 mice per treatment per genotype. (**B**) The intestine tissue were harvested at indicated timepoints from *Cebpd*^+/+^ and *Cebpd*^−/−^ mice and analyzed for 3-NT. The data are presented as average + S.E.M. of n = 7–9 mice per treatment per genotype.

iNOS (inducible nitric oxide synthase) or *Nos2* is involved in immune response and is known to be significantly induced by exposure of cells/tissues to IR^39–42^. *Cebpd*^−/−^ mice showed robust induction of *Nos2* expression in intestine by 10-fold, which is significant compared to respective *Cebpd*^+/+^ mice at 1 h post-irradiation. Both *Cebpd*^+/+^ and *Cebpd*^−/−^ showed elevated expression of *Nos2*, however it reduced about 25- and 22-fold at 4 h and to 2.4-fold and 1.6-fold at 1 day post-irradiation respectively. At day 3.5 post-irradiation, the *Nos2* transcript was induced by 2.6-fold *Cebpd*^−/−^ mice compared to 3-fold in *Cebpd*^+/+^ mice and was not significant (Fig. 5A).

This post-irradiation induction of *Nos2* results in increased nitric oxide levels, which react with the oxygen free radicals produced in the cells as a consequence of radiation-induced oxidative stress to form peroxynitrite, which causes nitrosylation of the cellular proteins^43,44^. The intestine tissues of *Cebpd*^+/+^ and *Cebpd*^−/−^ mice were compared for the expression levels of 3-nitrotyrosine (3-NT) by HPLC. In the sham group, the intestinal tissue extracts of *Cebpd*^−/−^ mice showed a 1.7-fold increase compared to *Cebpd*^+/+^ mice. Exposure to IR further showed significant increase in the 3-NT expression in the intestinal tissues of *Cebpd*^−/−^ mice compared to *Cebpd*^+/+^ mice and with respect to the sham controls (Fig. 5B).

### *Cebpd*-deficiency results in basal and IR-induced oxidative stress

**Figure 6:**
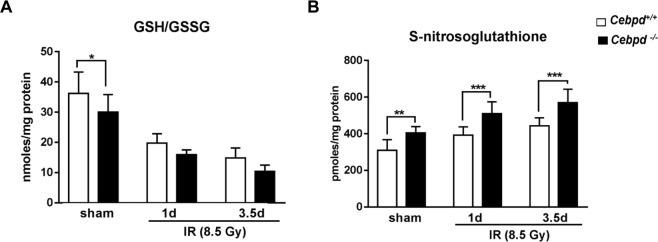
*Cebpd*^−/−^ mice display increased oxidation and nitrosylation of GSH. (**A**) The levels of oxidized to reduced GSH and (**B**) GSNO were measured in intestine tissues of *Cebpd*^+/+^ and *Cebpd*^−/−^ mice at indicated timepoints post-irradiation. The data are presented as average + S.E.M. of n = 7–9 mice per treatment per genotype.

Glutathione (GSH) is the global antioxidant which plays a critical role in maintaining the redox state of cells and detoxification of IR-induced oxidative stress^45,46^. We therefore examined the expression of GSH and its oxidized dimer glutathione disulfide (GSSG) in intestine tissues. The levels of GSH and GSSG were decreased by IR and were not significantly different between both genotypes (Supplementary Fig. 1). A decrease in the ratio of GSH/GSSG is considered a measure of oxidative stress. Interestingly, we found that in the sham group, *Cebpd*^−/−^ mice displayed a significant decrease in the GSH/GSSG compared to *Cebpd*^+/+^ mice (Fig. 6A). At days 1 and 3.5 post-irradiation, although there was a decrease in the GSH/GSSG ratio in both the genotypes, however these differences were not significant. In addition, GSH also acts a scavenger of nitric oxide to form S-nitrosoglutathione (GSNO)^47,48^. We examined in both *Cebpd*^+/+^ and *Cebpd*^−/−^ intestinal tissues and found that the GSNO levels were significantly elevated at basal levels as well as post-irradiation in *Cebpd*^−/−^ mice compared to *Cebpd*^+/+^ mice (Fig. 6B).

### *Cebpd*-deficient mice show IR-inducible upregulation of Claudin-2 and altered localization in intestine tissue

**Figure 7:**
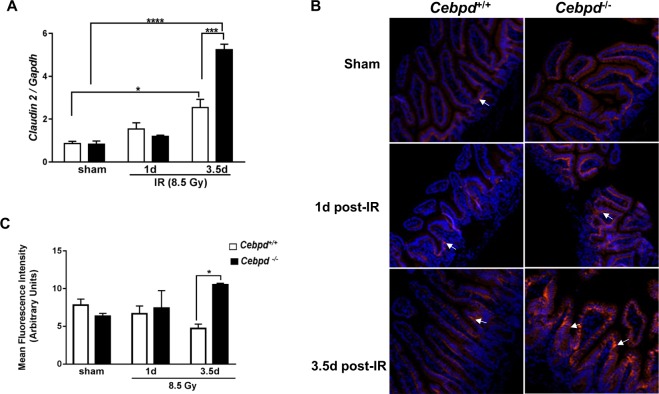
*Cebpd*^−/−^ mice show radiation-induced upregulation of Claudin-2 in the intestine. (**A**) The expression of *Cldn2* was analyzed in intestine tissues of *Cebpd*^+/+^ and *Cebpd*^−/−^ mice harvested at indicated timepoints post-irradiation. The values are expressed as fold changes normalized to unirradiated *Cebpd*^+/+^ control mice. The data are presented as average + S.E.M. of n = 7–9 mice per treatment per genotype. (**B**) Representative images of immunofluorescence staining of Claudin 2 in cross sections of the small intestine of *Cebpd*^+/+^ and *Cebpd*^−/−^ mice harvested at indicated timepoints post-8.5 Gy. (**C**) Quantification of Claudin-2 expression levels in intestinal crypts and villi at indicated time-points after exposure to IR. Values are presented as mean +SEM, n = 3 per genotype per group.

There were no significant changes between both the genotypes in the expression of *Cldn-2* in sham unirradiated controls and at day 1 post-irradiation. However at day 3.5 post-irradiation, *Cldn2* mRNA and protein levels were upregulated by 2-fold in the intestine tissues of *Cebpd*^−/−^ mice compared to that of *Cebpd*^+/+^ mice (Fig. 7A,C). Some of the other genes such as *Cldn4, Cldn11, Ocldn* and *Zo-1* did not any significant differences between both the genotypes either in the sham or at day 1 and 3.5 post-irradiation (Supplementary Fig. 2). Further immunofluorescence staining of intestine sections revealed that there were no significant differences in the expression of Claudin-2 in the sham or at day 1 post-irradiation in both the genotypes. The Claudin-2 expression was localized on the basal surface of the epithelial cells in the sham group and at day 1 post-irradiation in both *Cebpd*^−/−^ and *Cebpd*^+/+^ mice. At day 3.5 post-irradiation, the localization of Claudin-2 was found in the tight junctions between the epithelial cells as well on the luminal surface in *Cebpd*^−/−^ mice. In contrast the *Cebpd*^+/+^ mice showed the Claudin-2 expression on the basal surface of the epithelial cells (Fig. 7B).

### *Cebpd*-deficient mice show increased IR-induced *in vivo* intestinal permeability, increased endotoxemia and bacterial translocation

**Figure 8:**
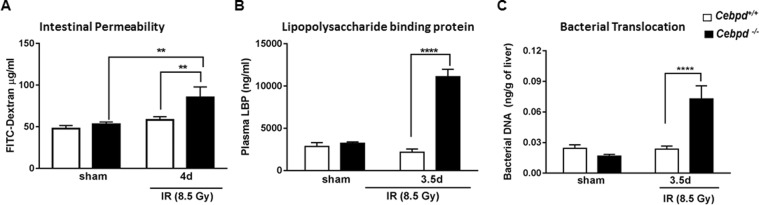
*Cebpd*^−/−^ mice show increased intestinal barrier disruption and bacterial translocation after exposure to IR. Blood, intestine and liver tissues were harvested at the indicated timepoints from *Cebpd*^+/+^ and *Cebpd*^−/−^ mice after exposure to IR. (**A**) Intestinal permeability was measured by analyzing plasma samples for the presence of FITC-Dextran, (**B**) Bacterial translocation was measured by amplification of 16S rRNA in liver tissues by real-time PCR and (**C**) LBP was measured in plasma samples by ELISA. All the data are presented as average + S.E.M. of n = 7–9 samples per treatment group.

As a functional consequence of Claudin-2 upregulation, next we determined whether irradiated *Cebpd*^−/−^ mice showed alterations in intestinal permeability. *Cebpd*^−/−^ mice showed a 2-fold increase in FITC-dextran levels in plasma compared to *Cebpd*^+/+^ mice at day 3.5 post-irradiation indicative of increased intestinal permeability (Fig. 8A). We further confirmed whether increased intestinal permeability led to a significant increase in the lipopolysaccharide-binding protein (LBP) in *Cebpd*^−/−^ mice after exposure to IR. There were no differences between both genotypes in the sham controls. However, at days 3.5 and 7 post-irradiation, compared to *Cebpd*^+/+^ mice, there was a 5-fold and a 3.7-fold increase in the plasma levels of LBP in *Cebpd*^−/−^ mice, indicative of bacteria in systemic circulation (Fig. 8B). These results correlated with a 2-fold increase in the amplification of 16S rRNA in liver tissues at day 3.5 post-irradiation in *Cebpd*^−/−^ mice compared to that of *Cebpd*^+/+^ mice, indicating translocation of bacteria from the intestine (Fig. 8C). All these results demonstrate that the increased leakiness of the gut in the *Cebpd*^−/−^ mice at day 3.5 post-irradiation leads to the onset of sepsis-like sequelae.

## Discussion

Exposure to the whole or substantial parts of the body to IR often result in life-threatening injuries, primarily to self-renewing tissues such as the hematopoietic and GI^1,3,6,7^. The main cause of lethality after exposure to IR is due to the intestinal bacteria that penetrate the defective mucosal barrier and are an important source of bacteremia. An increase in mucosal permeability occurs through a combination of disruption of epithelial tight junctions and insufficient replacement of the villus epithelium, due to cell death of intestinal progenitor cells in the crypts. Enhanced intestinal permeability, leading to bacterial and lipopolysaccharide translocation are characteristics of IR-induced multiple organ dysfunction syndrome^16,49^.

Previously, we reported that the increased lethality to IR exposure observed in *Cebpd*^−/−^ mice occurred due to increased thrombocytopenia, neutropenia and loss of intestinal crypts^32^. These very same processes are implicated as key hallmarks of sepsis^16,49^. In this study we show that *Cebpd*^−/−^ mice show increased damage to the intestinal villi and crypts at day 7 post 8.5 Gy. Further the loss of intestinal crypts at a dose of 10 Gy suggests a direct role for C/EBPδ in protection of intestinal crypt epithelial cells.

The IR-induced inflammatory response is initiated by the production of ROS/RNS that promote the induction of apoptosis and clonogenic cell death, activation of the transcription of several pro-inflammatory cytokines, chemokines, and growth factors in the microvascular and mucosal compartments, by the recruited immune cells and by enterocytes and residing cells, depending on the severity of tissue trauma^10,15,50,51^. The production of cytokines such as *Il-6* and *Tnf-α* is time-dependent usually peaking between 4–24 h post-irradiation with subsequent decrease to basal levels with 24 h to few days^50^. It is now realized that sepsis is associated with an “inflammatory storm”, which results in multi-organ damage/failure^16–18^. *Mcp-1* is one of the key chemokines that regulates migration and infiltration of monocytes/macrophages^34^, while *Cxcl1* is implicated in recruiting neutrophils that are frequently the first immune cells to enter an inflamed or infected tissue^35^. *Mif-1α* is an integral component of host inflammatory responses and is known to be induced by IR and is positively associated with sepsis^36–38^. In the present study, we found that intestines of *Cebpd*^−/−^ mice showed rapid upregulation of the pro-inflammatory cytokines, *Il-6* and *Tnf-α* at early timepoints post-irradiation. In contrast, the expression of chemokines such as *Mcp-1*, *Cxcl1* and *Mif1-α* were upregulated by days 3.5 and 7 post-irradiation compared to *Cebpd*^+/+^ mice.

Inducible nitric oxide synthase (iNOS) is expressed by infiltrating as well as resident activated macrophages in inflamed gastrointestinal tissue and is also stimulated by pro-inflammatory cytokines like *Il-6* and *Tnf-α* as well as by IR^42^. In response to IR exposure, the increased nitric oxide reacts with superoxide formed by the mitochondria to form peroxynitrite (ONOO^−^) which is a proxidant. Peroxynitrite reacts with the tyrosine residues in the cellular proteins and forms 3-NT^43,44^. Increased 3-NT causes increased radiation-induced intestinal toxicity and blocking or reducing 3-NT protects against radiation injury. In the present study, *Cebpd*^−/−^ mice show elevated levels of 3-NT at basal as well as post-irradiation compared to *Cebpd*^+/+^ mice.

GSH is the first line of defense for oxidative stress^45^. Decreased levels of GSH indicate decrease capacity to remove free radicals. The ratio of GSH/GSSG is a measure of redox state of the cell or tissue^46^. Interestingly, the expression of GSH/GSSG in the intestine tissues of *Cebpd*^−/−^ mice were significantly lower than that of *Cebpd*^+/+^ mice indicative of increased oxidative stress in the sham controls.

The elevated levels of NO produced are scavenged by the cellular antioxidant, GSH, resulting in the formation of GSNO^47,48^. GSNO is the nitrosylated form of reduced glutathione (GSH), responsible for its antioxidant cytoprotective action^47^. Although GSNO is shown to have a protective function in maintaining the epithelial barrier function^48^, we found that *Cebpd*^−/−^ mice expressed significantly elevated GSNO levels in the sham as well irradiated group. At very high concentrations, GSNO is converted to GSSG and NO and the released NO can react with superoxide present in the cell and generate increased peroxynitrite as described previously^43^. This could be a plausible mechanism for the increased 3-NT levels, as we have previously shown evidence for basal as well as IR-induced oxidative stress and mitochondrial dysfunction via reduced expression of GSH levels in *Cebpd*-deficient MEFs^33^. The increased mRNA levels of iNOS and increased expression of 3-NT and GSNO are indicative of overall increased nitrosative stress in the intestines of *Cebpd*^−/−^ mice compared to that of *Cebpd*^+/+^ mice.

The epithelial tight junctions form a barrier to the entry of allergens, toxins and pathogens across the epithelium into the interstitial tissue. The tight junction proteins, adherens junction and desmosomes seal the intercellular junctions of intestinal epithelial cells^52,53^. While the tight junctions function as a barrier from noxious molecules and a pore for the permeation of ions, solutes and water, the adherence junctions and desmosomes provide a strong adhesive bond between cells and in intercellular communication. One of the mechanisms via which inflammation promotes intestinal permeability is via the downregulation of tight junction proteins such as Occludin, junctional adhesion molecules (JAM), ZO-1, Claudins etc.^54–60^. In addition, ROS and RNS can promote the disruption of tight junctions and promote intestinal permeability^56,57^. Studies have shown that radiation injury led to downregulation of the tight junction proteins in the intestinal mucosa^54,55^. The upregulation of Claudin2 by IL-6 as well as TNF-α are known to cause an increase in intestinal permeability^60,61^. Similarly in this study, we found significantly elevated expression of *Il-6* and *Tnf-α*, which correlated with the upregulation of *Cldn2* in *Cebpd*^−/−^ mice at day 3.5 post-irradiation. Further studies in other inflammatory intestinal disorders have reported altered localization of Claudin 2 similar to that observed in irradiated *Cebpd*^−/−^ mice^62,63^. These results correlated with the increased *in vivo* intestinal permeability and elevated plasma LBP levels as well as bacterial translocation observed in irradiated *Cebpd*^−/−^ mice. *Cebpd*^−/−^ mice show increased inflammatory, oxidative and nitrosative stress that leads to increased intestinal permeability and bacterial translocation at day 3.5 post-irradiation, thus confirming that the increased mortality to radiation occurs due to underlying sepsis.

**Figure 9:**
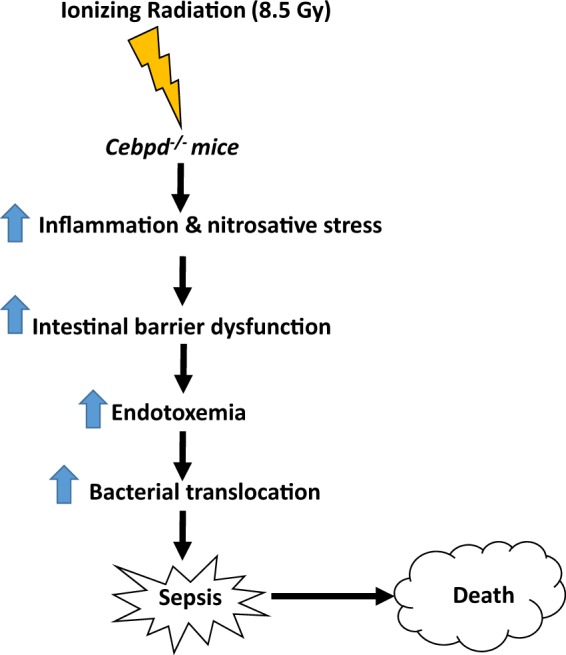
Schematic model depicting IR-induced increased inflammation and nitrosative stress that leads to increased intestinal permeability and bacterial translocation in *Cebpd*^−/−^ mice.

The present study uncovers a novel role for C/EBPδ in protection from IR-induced gut injury and underlying sepsis-mediated lethality by downregulating the IR-induced oxidative/nitrosative stress and inflammatory responses (Fig. 9). Further studies are warranted to elucidate whether blocking the IR-induced inflammation and oxidative/nitrosative stress can alleviate the lethality of *Cebpd*-deficient mice to IR. Overall these results may have relevance in terms of human exposure to IR either in accidental exposure or in the clinical setting. Recently we have described that C/EBPδ is essential to mediate the radioprotective functions of the potent radioprotector gamma-tocotrienol (GT3)^64^. We found that GT3 induces the C/EBPδ expression in the intestine and helps protect the *Cebpd-*WT mice, but was unable to impart protection to *Cebpd*-KO mice from radiation induced injury to the intestinal and hematopoietic systems. Therefore it can be speculated that agents that induce C/EBPδ expression may have the potential to protect normal tissue from radiation-induced damage to the intestine tissues in the clinical setting as well.

## Materials and Methods

### Ethics statement

This study was carried out in strict accordance with the recommendations in the Guide for the Care and Use of Laboratory Animals of the National Institutes of Health and approved by the Institutional Animal Care and Use Committee of the University of Arkansas for Medical Sciences.

### Animals

*Cebpd*-heterozygous breeder mice were backcrossed for more than 20 generations to the C57BL/6 strain background. Genotyping was done as described previously^65^. In all the studies, 10–12 weeks old subjects derived from heterozygous mating pairs and litter mate controls were used whenever possible. The animals were housed in the Division of Laboratory Medicine (DLAM, University of Arkansas for Medical Sciences, Little Rock, AR) under standardized conditions with controlled temperature and humidity and a 12 h day, 12 h night light cycle. Blood, intestine and liver tissues were harvested following isoflurane inhalation to minimize suffering and the animals were euthanized by cervical dislocation.

### Irradiation of mice

Irradiation was administered in a Mark I irradiator (J. L. Shepherd). Dose uniformity was assessed by an independent company (Ashland Specialty Ingredients) with radiographic film and alanine tablets. Alanine tables were analyzed by the National Institute of Standards and Technology (Gaithersburg, MD, USA) and demonstrated a dose rate of 1.14 Gy/min at 21 cm from the source. For each experiment the dose rate was corrected for decay.

### Assessment of villus height and crypt depth

Intestinal tissue sections stained with hematoxylin and eosin (H&E) were used to measure villus height and crypt depth using a computer-assisted image analysis platform (Imagescope ver 12.2.2.5, Leica Biosystems, e, MD, USA). Intestinal tissues fixed in Methyl–Carnoy’s fixed and embedded in paraffin were cut into 2–4 μm sections with a microtome. The slides with tissue sections were de-waxed by placing in an incubator overnight set at 60 °C, cooled down to room temperature, dipped into hematoxylin solution for 30 s, washed with deionized water, stained with 1% eosin solution, dehydrated with two changes in 95% and 100% alcohol for 30 s each, washed with xylene, and finally mounted with low viscosity Permount™ mounting media (Thermo Fisher Scientific, Grand Island, NY, USA). Mucosal villus height was measured from the tip to the base of each villus, and crypt depth was measured from the crypt base to the top opening. Images captured at 4x magnification were analyzed for crypt to villus measurements. 20 villi and crypts were respectively analyzed for villous height and crypt depth measurements on images at 20x magnification.

### Immunohistochemical staining for PCNA

Jejunums were collected from *Cebpd*^+/+^ and *Cebpd*^−/−^ mice 0 and 3.5 days post-TBI (0, 7.4, 8.5 and 10 Gy), after isoflurane anesthesia. The intestine tissues were fixed with methanol carnoy’s fixative (methanol: chloroform: acetic acid, 6:3:1) for 24 h, dehydrated, were embedded in paraffin in cross-sectional orientation. For staining, sections of 5-µm thickness were cut, dewaxed, rehydrated in PBS (10 mM sodium phosphate, pH 7.4; 140 mM NaCl).

Samples were probed with antibody to PCNA (FL-261)-sc7907 (Santa Cruz Biotechnology Inc., Dallas, TX) at 1:100 dilution in blocking buffer (2% goat serum, PBS). For control sections, blocking buffer contained blocking solution only (no primary antibody). The PCNA expression was detected with biotinylated anti-rabbit IgG (Vector Laboratories, BA-1000) at 1:400 dilution and developed with 3, 3′ diaminobenzidine tetrahydrochloride. Images for PCNA were captured at 20X magnification.

### Immunofluorescence staining for Claudin-2

The intestine tissues were fixed with methanol carnoy’s fixative (methanol: chloroform: acetic acid, 6:3:1) for 24 h, dehydrated, were embedded in paraffin in cross-sectional orientation. Sections were stained with rabbit anti-Claudin-2 (ab53032) (Abcam) at 1:50 dilution in blocking buffer (0.5% BSA, 0.05% Tween-20, PBS). Each section was probed and goat anti-rabbit IgG-AlexaFluor 594 (Invitrogen) at 1:400 dilution for 1 h at 37 °C and then rinsed three times with 0.05% Tween-20 in PBS. After staining, sections were counterstained with 4′,6-diamidino-2-phenylindol to visualize cell nuclei and were mounted under cover slips with Prolong Antifade kit (Invitrogen). Images for Claudin-2 and DAPI staining were acquired with an Olympus IX-51 inverted microscope (Olympus America) with a 10× objective, equipped with Hamamatsu ORCA-ER monochrome camera (Hamamatsu Photonics K.K.). For claudin-2 staining, a total of 5 fields of view per tissue section (10× objective), and the mean intensity was measured in each compartment. The average mean intensity of the field of view per tissue section was considered as a datapoint for statistical analysis. The data are presented as mean fluorescence intensity per field in n = 4 animals per timepoint. Slidebook 4.2 software (Intelligent Imaging Innovations, Inc.) was used for image capturing and analysis.

### Real-time PCR

Total RNA was purified from frozen tissue using RNeasy Plus Mini Kit (Qiagen, Valencia, CA, USA) as instructed by the manufacturer after homogenizing the samples in TRIzol^®^ Reagent (Life Technologies, Grand Island, NY, USA). cDNA was synthesized using a cDNA reverse transcription kit (Life Technologies, Grand Island, NY, USA) after treating the RNA samples with DNase I (Qiagen, Valencia, CA, USA). Predesigned Taqman assay for mouse genes: *Il-6*, Mm00446190_m1; *Tnf-α* Mm00443258_m1; *Cxcl1*, Mm04207460_m1; *Mcp-1*, Mm00441242_m1; *Mif-1α*, Mm01611157_m1; *Nos2*, Mm00440502_m1; *Cldn2*, Mm00516703_s1; *Cldn4*, Mm00515514_s1; *Cldn11*, Mm00500915_m1; *Ocldn*, Mm00500912_m1; *Zo-1*, Mm00493699_m1 and *Gapdh*, Mm99999915_g1 were obtained from (Life Technologies, Grand Island, NY, USA). *Gapdh* was used as an endogenous reference gene. Fold changes were calculated by normalizing to unirradiated WT sample using the standard 2^ΔΔ*Ct*^ method as described previously^23^.

### HPLC assays

High-performance liquid chromatography (HPLC) was used to quantify the reduced as well as oxidized glutathione (GSH, GSSG), *S*-nitrosoglutathione (GSNO) and 3-nitrotyrosine (3-NT). Approximately 20 mg of intestine tissue were homogenized in ice-cold phosphate-buffered saline. To precipitate proteins, 10% metaphosphoric acid was added to the homogenate and incubated for 30 min on ice. The samples were then centrifuged at 18,000 *g* at 4 °C for 15 min, and 20 µl of the resulting supernatants were injected into the HPLC column for metabolite quantification, while the pellet was used for protein analysis using BCA protein assay. The details for HPLC elution and electrochemical detection of free unbound GSH, GSSG, GSNO and 3-NT in proteins (hydrolyzed by 6 N HCl treatment) have been described previously described^66^.

### *In vivo* intestinal permeability assay

Intestinal permeability was measured in 20 mice (5 per group) as described by Biju *et al*.^67^. Briefly, 4 days after exposure to 0 and 8.5 Gy irradiation, the mice were anesthetized with isoflurane inhalation, a midline laparotomy was performed, and the renal artery and vein were ligated bilaterally. A 10 cm small intestinal segment, located 5 cm distal to the ligament of Treitz was isolated and tied off. One hundred microliters of 4-kDa fluorescein isothiocyanate conjugated dextran (FITC-dextran 25 mg/ml in phosphate–buffered saline) was injected into the isolated intestine using a 30 Gauge needle and the abdominal incision was closed. After 90 min, blood was collected from the retro-orbital sinus and plasma was separated by centrifuging at 4 °C, 8000 rpm for 10 min. The concentration of FITC-dextran was determined with a fluorescence spectrophotometer SpectraMax M2^e^ (Molecular Devices, CA, USA) at an excitation wavelength of 480 nm and an emission wavelength of 520 nm. Standard curves were prepared from dilutions of FITC-dextran in PBS to calculate FITC-dextran concentration in the plasma samples.

### ELISA assay

Plasma LBP level was measured using the mouse LBP ELISA kit (HK205) from Hycult Biotech, Uden, Netherlands. Briefly, blood was collected in EDTA coated tubes, centrifuged at 2000 rpm at 4 °C for 15 min and plasma samples were snap frozen and stored at −80 °C. One hundred μl of standard and plasma samples were loaded onto pre-coated 96-well plates, incubated for 2 h at room temperature. Biotinylated tracer antibody was incubated for 1 h at room temperature, developed against a streptavidin-peroxidase conjugate, and absorbance was measured at 450 nm. The concentration of LBP was determined against the standard curve. Values are expressed as nanogram LBP protein per ml plasma.

### Bacterial translocation

Bacterial translocation was determined as bacterial load in liver tissue and was quantified by real time PCR using the 16S rRNA gene consensus sequence. The total load of bacteria in the liver was determined using primer sequences to amplify the highly conserved sequence for a broad species consensus as reported elsewhere^67^. Livers were removed aseptically and homogenized immediately. Bacterial translocation was quantified by real-time PCR. Briefly, DNA was isolated from sterile livers harvested at baseline and at day 3.5 post-exposure to 8.5 Gy using a DNA purification kit (Promega, Madison, WI). Real-time PCR was performed using PowerUp SYBR green PCR master mix (Applied Biosystems, Foster City, CA) and 16S rRNA gene targeted primers, forward (5′-ACTCCTACGGGAGGCAGCAGT-3′) and reverse (5′-TATTACCGCGGCTGCTGGC-3′). Serially diluted bacterial genomic DNA was used to generate the standard curve. PCR-derived bacterial counts were expressed as nanogram bacterial DNA per gram mouse liver tissue.

### Statistical analyses

Statistical analysis was performed using GraphPad Prism 7.0 (GraphPad Software, San Diego, California). Data were expressed as mean ± S.E.M. unless otherwise specified. One-way ANOVA analysis with Tukey’s post analysis was used to study the differences among 3 or more means. Significance was determined at 95% confidence interval and p < 0.05 was considered statistically significant.

## Supplementary information

Supplementary Information

## Data Availability

Data or reagent described in this study will be made available upon request.
