# Supplementary material for: C/EBPδ protects from radiation-induced intestinal injury and sepsis by suppression of inflammatory and nitrosative stress
Source: Sci Rep. 2019 Sep 27;9:13953. doi: 10.1038/s41598-019-49437-x (PMC6764943; doi:10.1038/s41598-019-49437-x)
Supplement: Supplementary file 1 — Supplementary Information [file 41598_2019_49437_MOESM1_ESM.pdf]

C/EBPδ protects from radiation-induced intestinal injury and sepsis by suppression of inflammatory and nitrosative stress

Sudip Banerjee[1], Qiang Fu[1], Sumit K. Shah[1], Stepan Melnyk[2], Esta Sterneck[3], Martin Hauer-Jensen[1], and Snehalata A. Pawar[1*]

[1]Division of Radiation Health, Department of Pharmaceutical Sciences, College of Pharmacy, University of Arkansas for Medical Sciences, Little Rock, AR, 72205; [2]Arkansas Children's Research Institute, Little Rock, AR, 72202; [3]Center for Cancer Research, National Cancer Institute, Frederick, MD-21702

Running title: C/EBPδ-protects against radiation-induced intestinal injury and underlying sepsis by downregulation of inflammation and nitrosative stress

Keywords: C/EBPδ, ionizing radiation, inflammation, oxidative stress, nitrosative stress, intestinal permeability, Claudin-2, 3-nitrotyrosine, S-nitrosoglutathione, bacterial translocation, sepsis

***Correspondence:**

Snehalata A. Pawar, Division of Radiation Health, Department of Pharmaceutical Sciences, College of Pharmacy, University of Arkansas for Medical Sciences, Little Rock, Arkansas, 72205, Email: SAPawar@uams.edu, Phone: 501-686-5784, Fax: 501-686-6057

Conflict of Interest: None

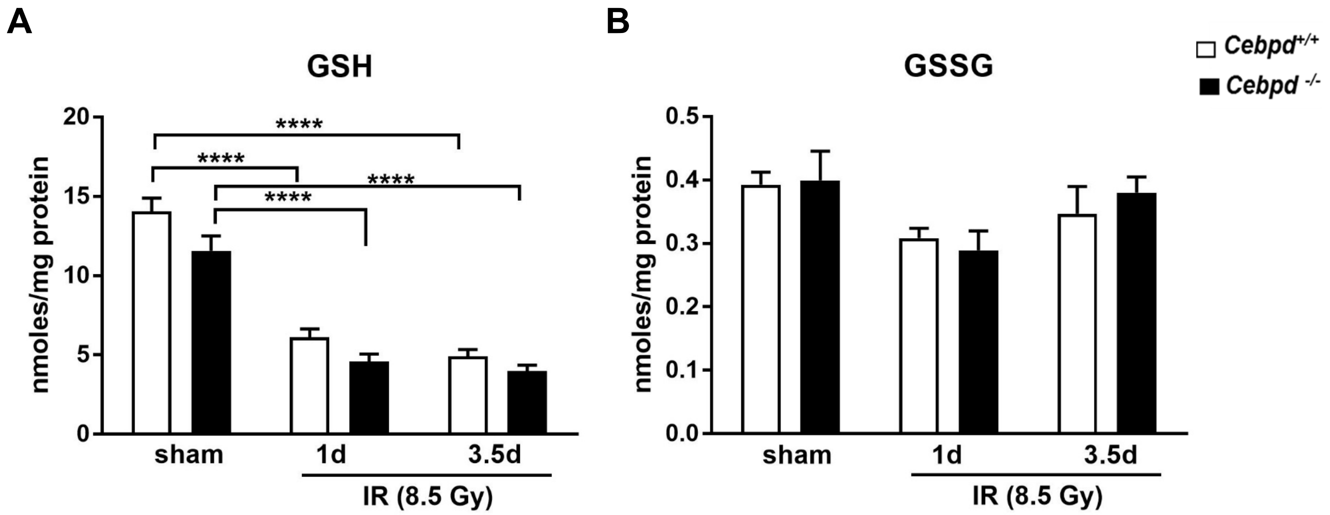
A
GSH
nmoles/mg protein
20
15
10
5
0
****
****
****
****
sham
1d
3.5d
IR (8.5 Gy)
B
GSSG
nmoles/mg protein
0.5
0.4
0.3
0.2
0.1
0.0
sham
1d
3.5d
IR (8.5 Gy)
Cebpd+/+
Cebpd -/-

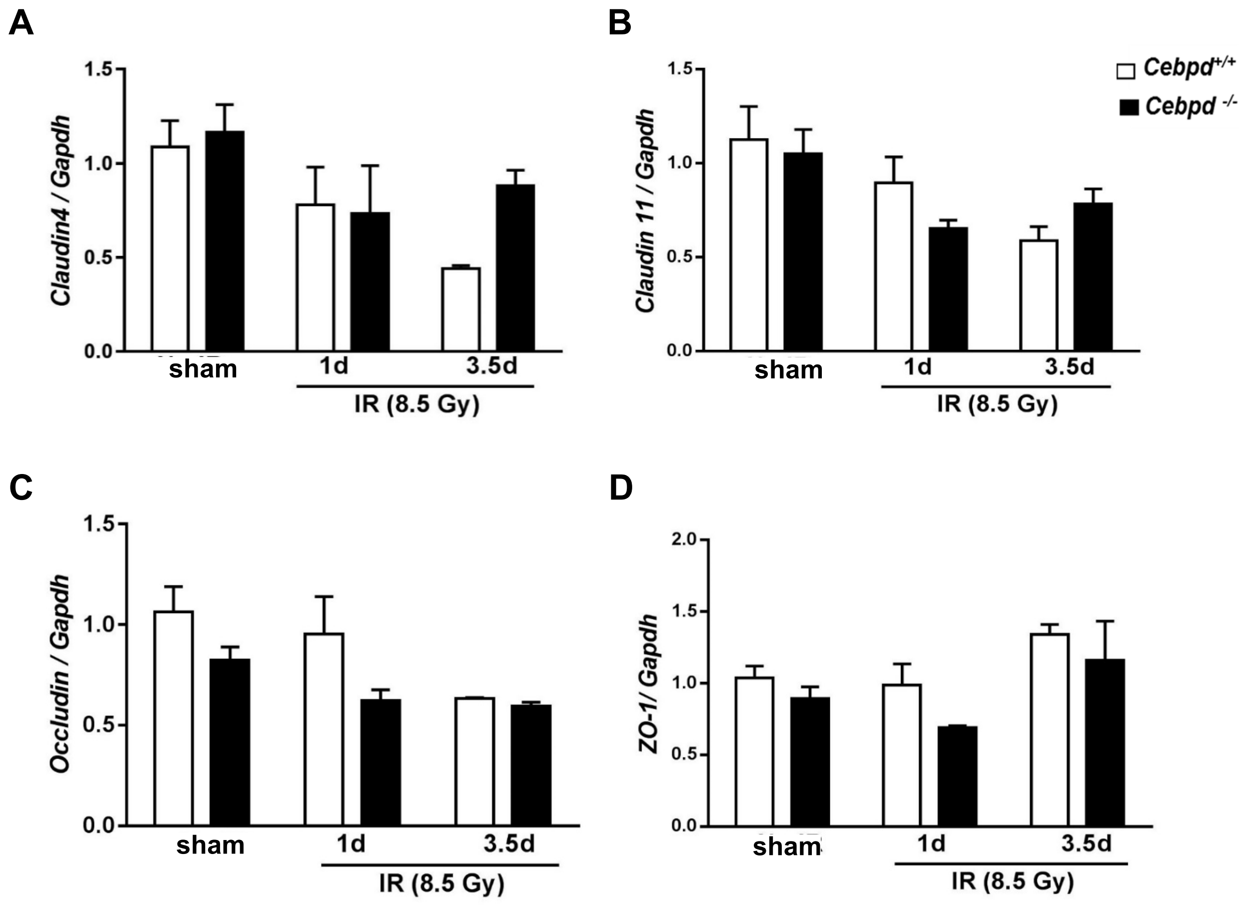
A
B
C
D
Claudin4 / Gapdh
Claudin 11 / Gapdh
Occludin / Gapdh
ZO-1/ Gapdh
1.5
1.0
0.5
0.0
2.0
sham
1d
3.5d
IR (8.5 Gy)
Cebpd+/+
Cebpd -/-
